# A systematic analysis of human lipocalin family and its expression in esophageal carcinoma

**DOI:** 10.1038/srep12010

**Published:** 2015-07-01

**Authors:** Ze-Peng Du, Bing-Li Wu, Xuan Wu, Xuan-Hao Lin, Xiao-Yang Qiu, Xiao-Fen Zhan, Shao-Hong Wang, Jin-Hui Shen, Chun-Peng Zheng, Zhi-Yong Wu, Li-Yan Xu, Dong Wang, En-Min Li

**Affiliations:** 1 Department of Pathology, Shantou Central Hospital, Affiliated Shantou Hospital of Sun Yat-sen University, Shantou 515041, China; 2 Department of Biochemistry and Molecular Biology, Shantou University Medical College, Shantou 515041, China; 3 Department of Oncology Surgery, Shantou Central Hospital, Affiliated Shantou Hospital of Sun Yat-sen University, Shantou 515041, China; 4 Institute of Oncologic Pathology, Shantou University Medical College, Shantou 515041, China; 5 College of Bioinformatics Science and Technology, Harbin Medical University, Harbin 150000, China.

## Abstract

The lipocalin proteins (lipocalins) are a large family of small proteins characterized by low sequence similarity and highly conserved crystal structures. Lipocalins have been found to play important roles in many human diseases. For this reason, a systemic analysis of the molecular properties of human lipocalins is essential. In this study, human lipocalins were found to contain four structurally conserved regions (SCRs) and could be divided into two subgroups. A human lipocalin protein-protein interaction network (PPIN) was constructed and integrated with their expression data in esophageal carcinoma. Many lipocalins showed obvious co-expression patterns in esophageal carcinoma. Their subcellular distributions also suggested these lipocalins may transfer signals from the extracellular space to the nucleus using the pathway-like paths. These analyses also expanded our knowledge about this human ancient protein family in the background of esophageal carcinoma.

The lipocalin proteins (lipocalins) are a large family of small extracellular proteins with 100–300 amino acid residues. They are found in both prokaryotes and eukaryotes^1^. Lipocalins typically transport or store small biological compounds, including vitamins, steroid hormones, odorants, and various secondary metabolites. The lipocalin genes show a conserved exon/intron arrangement^2^. Though the family demonstrates considerable diversity even at the protein sequence level, most lipocalins share one or three conserved sequence motifs, called structurally conserved regions (SCRs)^3^. Belying their sequence dissimilarity, the crystal structures of lipocalins are highly conserved and similar, comprising an eight-stranded hydrogen-bonded antiparallel beta-barrel, which acts an internal ligand-binding site^4^. The definition of human lipocalins has been expanded on the basis of sequence or structural similarity^5^. The lipocalins are conserved during the species evolution, and new members have been discovered in plants and bacteria^6^,^7^.

After more than 20 years of analyses, it has come to be generally believed that lipocalins play important roles in normal human physiological activities and in diseases^8^. Because of its antioxidant and anti-inflammatory activity, *Apolipoprotein D* (*APOD*) is upregulated in the aging human brain and elevated levels of APOD play a protective role in a large number of neurologic disorders, such as Alzheimer’s disease, schizophrenia, and stroke^9^. *LCN13* is expressed in multiple tissues, including those of the liver, pancreas, epididymis, and skeletal muscle. Obesity is associated with a downregulation of *LCN13* expression and lower levels of circulating LCN13. *LCN13* therapy enhances insulin sensitivity in adipocytes and improves glucose intolerance and hepatic steatosis^10^. *LCN1*, also called *tear lipocalin* (*TLC*), binds to macromolecules, which regulate tear viscosity, the binding and release of lipids, and endonuclease inactivation of viral DNA^11^. Hepatic overexpression of *apolipoprotein M* (*APOM*) in low-density-lipoprotein-receptor-deficient mice has been shown to lead to an approximately 70% reduction in atherosclerosis^12^.

Lipocalins play important role in the innate immune response to bacterial infection^13^. Bacterial pathogens usually obtain iron from the host by production of siderophores, which are small high-affinity iron chelating compounds, in order to survive and grow. As a defense, the host produces siderocalins, which limit pathogen growth by intercepting siderophores, preventing the delivery of iron to the pathogen. Siderocalins are a siderophore-binding subset of the lipocalins, and they include *LCN1*, *LCN2*, and *FABP*. Siderocalins have been identified in humans and many other mammals, linking the crosstalk between inflammation and cancer^14^.

In recent years, the functional roles of lipocalins in human cancers have been determined and published, which has drawn even more attention^15^. *LCN2* (*lipocalin-2*), also known as *neutrophil gelatinase-associated lipocalin* (*NGAL*), is overexpressed under many other pathologic conditions, including cancer. It is frequently associated with tumor size, stage, and invasiveness. Cumulative experimental results have demonstrated that *LCN2* has multiple functions in various cancers, including inhibition of apoptosis, stimulation of proliferation, and promotion of the epithelial-to-mesenchymal transition (EMT). LCN2 stabilizes the proteolytic enzyme matrix metalloprotease-9 (MMP-9) by forming a heterogeneous complex, thereby preventing autodegradation and promoting metastasis of cancer cells^16^. Another lipocalin associated with carcinoma is *glycodelin*, also called *PAEP* (*progestagen-associated endometrial protein*). It is involved in cell recognition and epithelial differentiation. Glycodelin reduces carcinoma cells growth both *in vitro* and *in vivo*, suggesting it acts as a tumor suppressor in breast cancer^17^. In breast cancer, APOD inhibits translocation of phosphorylated MAPK into the nucleus, reducing the proliferative activity of cancer cells^18^.

Though several reports have described the sequence, structure, and evolution of lipocalins in the past ten years, these reviews and analyses have specific limitations^19^,^20^,^21^. One weak point is that while the lipocalins have bene collected from different species, they have not included the new lipocalins identified in recent years. The other is that lipocalins have not been analyzed under a specific human pathological condition. In this study, human lipocalin proteins were collected. Their protein-protein interaction network (PPIN) was constructed, and their expression levels in esophageal carcinoma, including esophageal adnocarcinoma (EAC) and esophageal squamous cell carcinoma (ESCC) were integrated into the PPIN.

## Results

### Analysis of human lipocalin protein sequences and structures

**Figure 1:**
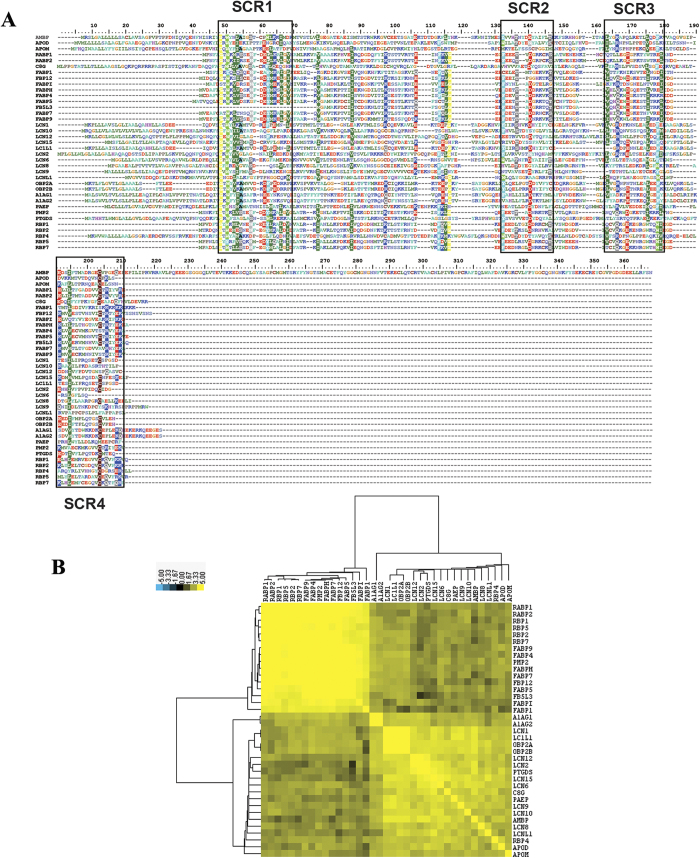
(**A**) Protein sequence alignment of human lipocalins. The four structurally conserved regions (SCRs) are indicated in four blocks. (**B**) Cluster of sequence similarities, indicating human lipocalins could be divided into two subgroups.

Currently, 37 lipocalins have been found in human genome. Information regarding human lipocalins is shown in Table 1. The alignment of lipocalin protein sequences is shown in Fig. 1A, with four structurally conserved lipocalin regions (SCRs) are clearly indicated (Supplementary Figure S1). AMBP has the longest sequence length with 352 amino acids, while FABP1 has the shortest sequence length (127 amino acids). The protein sequences of lipocalins were found to be less similar to each other than expected, without any obvious large conserved region. Every pair of lipocalins was clustered based on their sequence similarities were shown using a matrix (Fig. 1B). Results indicated that the lipocalins could be divided into two groups based on their protein sequence similarities. The first group contained RABPs (*RABP1* and *RABP2*), RBPs (*RBP1*, *RBP2*, *RBP5*, and *RBP7*), FABPs (*FABP1*, *FABP5*, *FABP7*, *FABPH*, *FABPI*, *FBP12*, and *FB5L3*), and *PMP2*. The second group contained A1AGs (*A1AG1* and *A1AG2*), LCNs (*LCN1*, *LCN2*, *LCN6*, *LCN8*, *LCN9*, *LCN10*, *LCN12*, and *LCN15*), OBP2s (*OBP2A* and *OBP2B*), *LC1L1*, *PTGDS*, *LCNL1*, *APOD*, *APOM*, and *PAEP*. This suggested that lipocalins might have evolved in two different ways, which may be useful in investigations of their biological functions.

Lipocalins are characterized by conserved protein tertiary structure^22^. The three-dimensional (3D) structures of lipocalins for which such information is currently available are listed in Supplementary Table S1. The 3D structures of two grouped lipocalins were compared to assess the classification of lipocalins based on their protein sequence similarities. In the first group, which contains RABPs, RBPs, and FABPs, only a half of a β-barrel with four antiparallel β-sheet strands was conserved. In the second lipocalin group, a whole conserved β-barrel made of a cylindrically closed β-sheet of eight antiparallel strands was conserved (Supplementary Figure S2).

### Functional enrichment analyses of lipocalins

**Figure 2:**
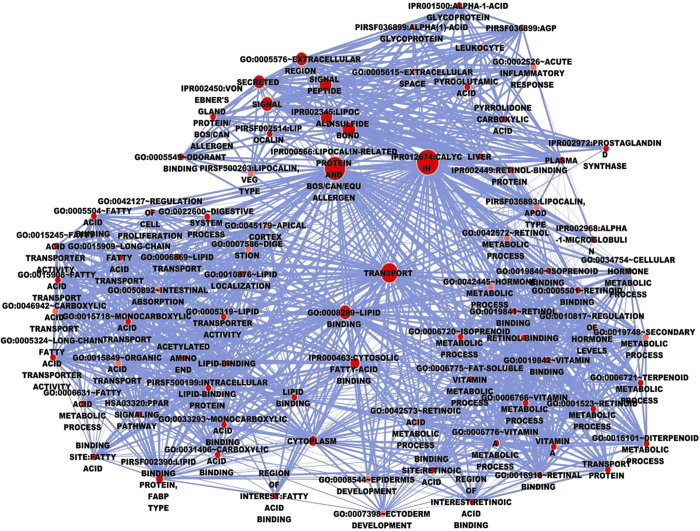
Functional categories of the lipocalins were visualized using the Enrichment map plugin of the Cytoscape. Significant functional terms are represented by one node with its size indicating the significance of the enrichment (*P*-value). Edges indicate gene overlap between nodes and thickness indicates the number of overlapping enriched genes.

To gain a full view of their potential functions and other important characteristics, the lipocalins were annotated using the Functional Annotation Chart and visualized using the Enrichment Map plugin in Cytoscape. As shown in Fig. 2, each node represented one functional annotation term. The more significant of the category, the deeper the color of the node. Nodes containing more enriched genes were larger. Edge width was here defined using the overlap coefficient between these categories (overlap coefficient cut-off 0.6). The more shared genes there were between two nodes, the wider the edge. Except 52 terms from Gene Ontology (GO) categories, the Functional Annotation Chart results also included 34 other terms from the following annotation categories, 9 INTERPRO, 18 SP_PIR_KEYWORDS, 6 UP_SEQ_FEATURE and 1 KEGG_PATHWAY. These results provide more information than the GO enrichment alone. The most significant enriched term was an InterPro annotation “IPR012674:Calycin.” InterPro is a database that provides functional analysis of protein sequences by classifying them into families and predicting the presence of domains and important sites^23^. Calycins form a large protein superfamily and share similar beta-barrel structures. This suggests that the conserved structure is the major reason why the lipocalin protein family has been conserved for so long. The three most enriched entries in Gene Ontology (GO) molecular function were “GO:0008289~lipid binding,” “GO:0005501~retinoid binding,” and “GO:0019840~isoprenoid binding,” which are associated with the three most well-known lipocalin ligands. Four lipocalins (*RBP4*, *FABP3*, *FABP4*, *FABP1*, and *FABP7*) were found to be involved in cell growth, and these were enriched in “GO:0042127~regulation of cell proliferation.” The top SP_PIR_KEYWORDS enrichment term was “transport,” indicating the main function of lipocalins. Here “hsa03320:PPAR signaling pathway,” the only term found in the KEGG_PATHWAY category, was associated with 6 genes (*FABP3*, *FABP4*, *FABP1*, *FABP2*, *FABP7*, and *FABP5*).

The GO-enriched results from WebGestalt also suggested that the lipocalin family was mostly involved in the metabolism of small molecules, such as lipids, retinoids, and vitamins (Supplementary Figure S3).

### Description of lipocalin co-expression PPIN and association with changes in esophageal carcinoma

**Figure 3:**
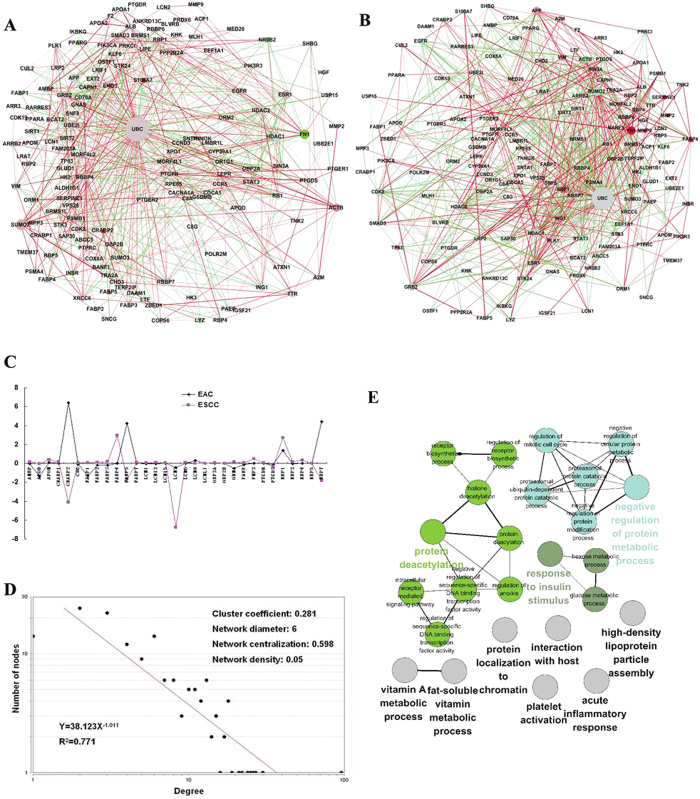
(**A**-**B**) The human lipocalin protein-protein interaction network and the lipocalins’ changes in expression in esophageal adenocarcinoma and esophageal squamous cell carcinoma are shown. The lipocalins are shown using triangles, and their interacting proteins are shown in circles. Red indicates upregulation and green indicates downregulation. The size of the node indicates the degree (the number of its interacting proteins) of the node. Bigger nodes have higher degrees. The connection between two nodes is indicated by an edge. Red edges indicate positive correlations in the expression of two proteins, and green edges indicate negative correlations. Correlation strength is shown by the edge width. (**C**) Expression levels of lipocalins in esophageal adenocarcinoma and esophageal squamous cell carcinoma. (**D**) The power law distribution of the node degree network and analysis of other network parameters. (**E**) Functional map of the lipocalins PPIN. Functionally grouped network with GO terms are represented as nodes, which were linked based on their kappa score level (≥0.3), suggesting overlapped enriched genes. The similar GO terms were labeled in the same color.

A full screening of the lipocalins’ interactions with other proteins was performed to determine how they affect cellular activity. This may provide important clues of their functions. The PPI dataset from both acknowledged HPRD and BioGRID databases provided credible original data for subsequent analysis. The lipocalin PPIN was generated by mapping the lipocalins to the parental PPI network to extract the proteins that interacted directly and all their interactions, forming a sub-network for lipocalins containing 151 nodes and 569 edges (Fig. 3). Currently, the interactions of 23 human lipocalin proteins have been reported. An esophageal carcinoma expression profile GSE26886, containing clinical samples from normal esophageal squamous epithelium, esophageal adenocarcinoma (EAC), and esophageal squamous cell carcinoma (ESCC), was analyzed to determine the expression trends of lipocalins and the proteins with which they interact. The fold-changes of these proteins in EAC and ESCC and other important parameters were integrated into the PPIN (Fig. 3A,B). In Fig. 3A,B, the color of each node indicates the level of expression. The gradient from red to green indicates upregulation through downregulation, all relative to normal esophageal tissue. The size of the node indicates the degree of the node (the number of proteins with which it interacts directly). The bigger nodes indicate higher degrees of interactions, connecting with more proteins. Every two interacting nodes are linked by an edge. The correlations in the levels of expression of any two interacting proteins are here treated as edge weight. Red edges indicate positive correlations in the expression of two interacting proteins, and green edges indicate the negative expression correlation. The strength of the correlation is indicated by the width of the edge.

The expression values of 31 out of 36 lipocalins were found in esophageal carcinoma expression profile GSE26886. The trends in the expression of many lipocalins in both EAC and ESCC remained highly consistent (Fig. 3C). Some lipocalins showed significant changes in this esophageal carcinoma expression profile, and even the contrast trends were observed in EAC and ESCC. For example, FABP5 was upregulated 4.23-fold in EAC, but downregulated 1.68-fold in ESCC. CRABP2 was overexpressed in EAC by 6.38-fold and downregulated 4.14-fold in ESCC. LCN2 was significantly decreased 6.78-fold in ESCC.

### Lipocalin PPIN topology parameters

Real biological networks (e.g. PPIN) are distinguishable from random networks by their distinguishing topological characteristics. The power law of node degree distribution is one of most important criteria^24^,^25^. As shown in Fig. 3D, the distributions of node degree approximately followed power law distributions, with an R^2^ = 0.771. Like many other co-expression PPI networks, they exhibited scale-free topology with R^2^ values above 0.6^26^,^27^. As the node degrees increase, the average clustering coefficients declines continuously, indicating which node degree distributions fit the power line curve best. Other network parameters, including cluster coefficient, network diameter, network centralization and network density, are also shown in Fig. 3D. These results suggested that the lipocalin PPIN characterizes scale-free, small world. The illustration of four other important network topology parameters, including closeness centrality, topological coefficients, neighborhood connectivity distribution and average clustering coefficient distribution, are shown in Supplementary Figure S4.

### Functional annotation map of lipocalin PPIN

To assess cellular activities related to lipocalins through their protein interactions within the PPI network, the enriched GO “Biological Process” terms for total proteins in the lipocalin PPIN were also analyzed in network format. A functional annotation map containing 19 GO terms was generated. In this map, proteins are represented as nodes according to their enriched GO terms, with the edges connecting the GO terms indicative of proteins share the same enriched GO terms (Fig. 3E). Several GO terms that had not shown GO enrichment during the previous process were discovered using the functional annotation of lipocalins PPIN, including “negative regulation of lipoprotein oxidation,” “phosphatidylcholine biosynthetic process,” “intestinal absorption,” and “digestive system process.”

### Correlations in the expression of lipocalins in esophageal carcinoma

**Figure 4:**
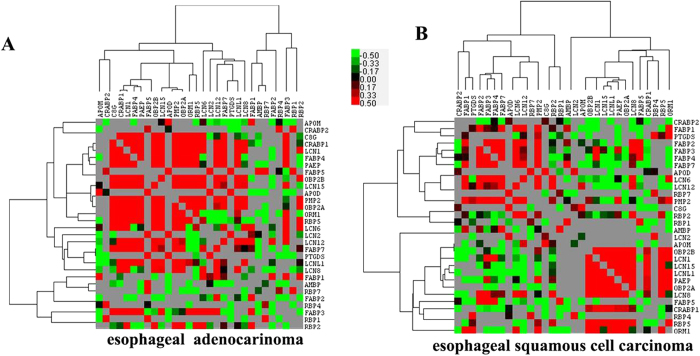
Co-expression patterns of lipocalins in esophageal adenocarcinoma and esophageal squamous cell carcinoma.

To gain insight into whether there are co-expression pattern for lipocalins in esophageal carcinoma, the expression correlation of lipocalins in EAC and ESCC were analyzed using the Pearson correlation, and then were also clustered and visualized (Fig. 4). Several pairs of significant correlations were found in the heatmap (Table 2). These results suggested that some lipocalins are co-expressed in esophageal carcinoma and they might co-operate for certain biological functions.

### Subcellular layers of lipocalins PPIN

**Figure 5:**
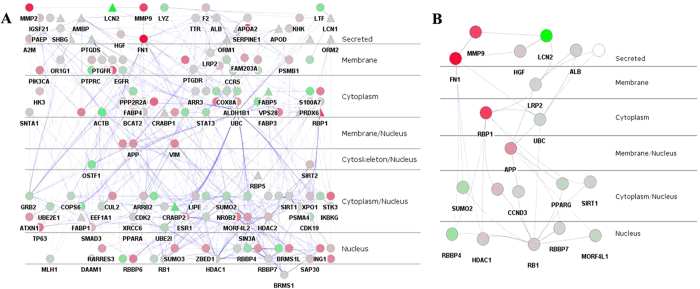
(**A**) Subcellular layers illustrating the lipocalins PPIN. (**B**) 20 possible paths from LCN2 to RB1 with integrated with their subcellular localization.

The appropriate subcellular localization and translocations of proteins are crucial to their functionality. Their functions include complex formation, signal transduction, protein modification, and disease^28^. In a network, nodes were re-distributed by Cerebral plugin according to their subcellular localization without changing their interactions. The lipocalin PPIN was here divided into 7 layers: secreted, membrane, cytoplasm, secreted/nucleus, membrane/nucleus, cytoplasm/nucleus, and nucleus (Fig. 5A). These results suggest that lipocalins can transfer cell signals of themselves or their binding complexes from the extracellular space to the intracellular space and nucleus, possibly through the noncanonical pathways.

To further illustrate the strength of this kind of analysis, the shortest path algorithm was used to find the possible shortest path from LCN2 to RB1 (retinoblastoma 1) and identify the linking proteins between LCN2 and RB1. RB1 is involved in many cellular pathways and acts as a transcriptional regulator. It can bind several transcription factors^29^. The 20 shortest paths from LCN2 to RB1 were found (Table 3); all were 4 in length. These proteins in the paths were distributed according to their sub-cellular localizations (Fig. 5B). Results confirmed that APOD can move into the nucleus after lipopolysaccharide (LPS) treatment^30^. The shortest paths from APOD to another transcription factor TP63 were also analyzed and 17 were found (Supplementary Figure S4 and Table S2).

## Discussion

Lipocalins are identified in various organisms, such as bacteria, plants, arthropods, and vertebrates^2^,^3^,^4^,^5^. An increasing number of sequences with lipocalin conserved domain are found in protein databanks. The amino acid sequences of lipocalins are quite diverse, and low levels of sequence identity, even below 20%, were found between the overall sequences among some members of the family. Despite the low level of sequence similarity, the tertiary structures of lipocalins are strongly preserved^31^. Although a great deal of attention has been paid to the lipocalin family across species, the lipocalins in *Homo sapiens* have not yet been reviewed in a systematic way, nor integrated with their expression data as they relate to human cancer. In this study, the human lipocalins were analyzed alongside their PPIN and their expression trends in esophageal carcinoma.

As in previous reports, the human lipocalins also contains the typical lipocalin domain. It has been reported that lipocalins across species contain three SCRs^19^. However, four SCRs were here found in human lipocalins^21^. This suggested that human lipocalins are more conserved than homologous lipocalins from other species. These human lipocalins could be divided into two groups based on the similarities of their protein sequence. This was also confirmed from the structural point of view by comparison of three-dimensional structures of these two grouped lipocalins. This suggested that human lipocalins might have evolved in two different ways, providing raw material for their functional innovation. These results also provided important clues to explore their different expressions and functions^32^.

To gain a full insight into the functions and characters of lipocalins, the Functional Annotation Chart from DAVID bioinformatics was used to annotate them. More than 40 annotation coverages were performed to increase the analytic power, allowing the investigators to analyze their genes from many different biological perspectives in a single space. Results showed the enrichment of lipocalins to be involved in functions related to binding and transporting. The functional terms not from the GO are an important additional information for GO. Other potential functions may be revealed in the future. These functional terms could be used to explain the multiple molecular mechanisms of lipocalins. For example, the enriched “PPAR signaling pathway” contained 6 lipocalins. It has been suggested that this pathway is essential to the regulation of cellular differentiation, development, and metabolism (carbohydrate, lipid, protein), and tumorigenesis of higher organisms^33^,^34^,^35^. These results provide links between lipocalins with human diseases and clues that can be used to assess possible functions of lipocalin.

Accumulated studies have shown that an integrative analysis of gene expression and PPIN can provide deep insights into the molecular mechanisms of diseases, or specific genes^36^,^37^. A PPIN was described for human lipocalins based on their direct proteins interactions. This is the first time that a human lipocalin PPIN has been presented showing their all known protein interactions. This lipocalin PPIN contained 151 proteins, including 23 lipocalins. Though this is a small, specific PPIN, the topological parameters, especially the power-law degree distribution, indicated that it is also a true biological network, characterized both small-world and scale-free. Lipocalins are characterized by multiple molecular recognition properties, including binding to their cell surface receptors. Many lipocalin receptors have been identified^38^. In the PPIN, the lipocalin receptors were easier to find than by searching the references one by one. *LCN2* is important gene. It promotes cancer cell metastasis and invasion in esophageal carcinoma, which transfers iron by snatching siderophores through its receptor, LRP2. Our previous study has identified a novel splicing variant of LCN2 receptor in ESCC. Both NGAL and its receptor are overexpressed in ESCC^39^,^40^. Esophageal cancer is the sixth most common fatal human cancer in the world, and the histological type of squamous cell carcinoma is one of the most common cancers in the Chinese population^41^,^42^. In this study, the expression data of lipocalins and their interacting proteins were integrated into lipocalin PPIN, to indicate their possible co-expression in esophageal carcinoma. Several lipocalins showed significant changes in esophageal carcinoma, suggesting that expression of these lipocalins might be correlated to the progression of esophageal cancer. Consistent with the results in this study, many lipocalins have been found to be dysregulated in esophageal carcinoma. *FABP5* is related to radiosensitivity of ESCC cell line TE-11, with a high degree of DNA methylation within its promoter region in three ESCC cell lines (TE-1, TE-2 and TE-10)^43^,^44^. The expression of *LCN2* was visibly decreased in the GSE26886 esophageal carcinoma expression data, which contradicts previous results. It was previously reported that both LCN2 and its receptor are upregulated in the Chinese ESCC clinical samples and can serve as independent prognostic factors for ESCC^40^. This difference might be attributable to the different sources of esophageal carcinoma clinical samples; the clinical samples of GSE26886 came from Germany. Another GEO dataset GSE45168, was designed to analyze the esophageal cancer clinical samples collected in China. In this case, LCN2 was found to be upregulated with 3.01-fold in GSE45168.

It was here presumed that the biological effects of lipocalins can become more pronounced through the cascades of protein-protein interactions. The lipocalin PPIN was annotated using GO in a network format, showing that this PPIN involves various biological entities, closely related to the currently known functions of lipocalins. However, it was here believed that this kind network functional annotation for PPIN could be expanded when more lipocalins directly interacting proteins that are identified in future.

Another interesting finding in this study is the co-expression pattern of lipocalins in esophageal carcinoma. For example, *LCN2* is significantly co-expressed with *RBP4* in both EAC and ESCC. Several reports have used the detection of both *LCN2* and *RBP4* as biomarkers of risk of disease. It is here suggested that circulating levels of *LCN2* and *RBP4* are positively correlated with carotid IMT and subclinical atherosclerosis in type 2 diabetes^45^. Significant elevation in serum concentrations of *LCN2* and *RBP4* has been observed in pancreatic cancer patients^46^. These results highlight a possible role of the co-expressed lipocalins in the pathogenesis and their possible interplay in diseases.

Subcellular localization is one type of important information that can indicate the participation of proteins in the cellular activities at the subcellular level^47^. Usually, cellular signaling is transduced by the certain flows or cascades of PPI, which are distributed in several of subcellular localizations. In this study, subcellular localization information was incorporated into the lipocalin PPIN, generating biologically intuitive pathway-like layouts in the network. It was here presumed that the lipocalins not only transported small molecules into the cell, but also transferred extracellular signals into the cell and even the nucleus through the cascades of protein-protein interactions. Several lipocalins are able to translocate into nucleus. It has suggested that one lipocalin, *CRABP2* (cellular retinoic acid-binding protein 2), transports retinoic acid into the nucleus to regulate transcription of target genes with heterodimeric nuclear receptors *RAR*(*α, β, γ*)^48^. Do *et al*. found that, under normal conditions of NIH/3T3, APOD was mainly perinuclear but it accumulated in the cytoplasm and nucleus under these stress conditions. The nuclear APOD appears to have been derived from the secreted protein. Do *et al*. supposed that it might act as an extracellular ligand transporter or transcriptional regulator depending on its location^30^. For this reason, it is credible that the lipocalin PPIN may directly or indirectly affect the signal cascades through the flow of extracellular-membrane-cytoskeleton/cytoplasm-nucleus, causing many different biological effects. To find possible paths from extracellular to nucleus, the possible shortest paths from LCN2 to RB1 were analyzed. A total of 20 paths between LCN2 and RB1 were found. According to their subcellular locations, most of these paths follow the flow from extracellular to cytoplasm to the nucleus. It is possible that the lipocalins can affect signal transduction and subsequent biological effects.

In summary, the current findings may facilitate a more comprehensive understanding of human lipocalins. The PPIN outlined here was integrated with the expression data from human cancer and the functional enrichment annotation. These analyses also expand our knowledge of this ancient protein family. The current study also provided a workflow to analyze a protein family through high-throughput experiments.

## Materials and methods

### Human lipocalin protein sequence collection

Human lipocalins were collected based on searches in the National Center for Biotechnology Information database (http://www.ncbi.nlm.nih.gov/protein), and the proteins of these genes were confirmed to contain the lipocalin domain through a query in UniProt protein database (http://www.uniprot.org/). The FASTA format of the protein sequences was retrieved from Uniprot database for future analyses.

### Protein sequence alignment and comparison of structures

The human lipocalin protein sequence alignment was performed using ClustalW embedded in BioEdit software (http://www.mbio.ncsu.edu/bioedit/bioedit.html) to indicate the conserved regions. The alignment was also carried out at http://www.ebi.ac.uk/Tools/msa/clustalw2/ to obtain the percent identity matrix result, which contained features similar to those of every two protein sequences. To visualize the results, the matrix was log-transformed, clustered using Cluster 3.0 software and viewed in the TreeView^49^. To further illustrate the conservation of lipocalins at three-dimensional (3D) structure level, the 3D structures of lipocalins available as of May 2015 were retrieved from PDB database (http://www.rcsb.org/pdb/home/home.do) and compared using the PDBeFold program (http://www.ebi.ac.uk/msd-srv/ssm/).

### Functional Annotation Chart of lipocalins

To better understand the functional classification and correlation of the lipocalins, an enrichment analysis was performed using Functional Annotation Chart in DAVID bioinformatics (http://david.abcc.ncifcrf.gov/). This system can identify over-represented biological terms associated with a given gene list. Functional Annotation Chart covers more than 40 annotation categories, including Gene Ontology (GO) terms, protein-protein interactions, protein functional domains, disease associations, pathways, sequence features, homology, and gene functional summaries. Terms from the Functional Annotation Chart that were significantly enriched were visualized using the Cytoscape Enrichment Map plugin (*P* < 0.05)^50^. To compare and confirm the results shown on the Functional Annotation Chart, the lipocalin genes were also enriched in WebGestalt at http://bioinfo.vanderbilt.edu/webgestalt/.

### Protein-protein interaction network (PPIN) construction

The newest versions of validated human protein-protein interaction datasets were downloaded from both HPRD (http://www.hprd.org/) (Release 9) and BioGRID (http://thebiogrid.org/) (Release 3.2.107), which are derived from studies of both low- and high-throughput experimental results^51^,^52^. These two datasets have been widely used in studies of disease involving human PPI networks, and their reliability has been assessed. In this study, the non-redundant interactions of *Homo sapiens* species from these two datasets were integrated manually. They contained 18,595 unique proteins and 174,552 interactions and were used as the parental PPIN. Cytoscape software was used for construction, visualization, and analysis of PPIN^53^. In Cytoscape, a PPIN is illustrated as a graph with the nodes as the proteins and the edges representing their interactions. A lipocalin PPIN was constructed. It contains both the lipocalins and their direct PPI neighbors and the interactions between these proteins. The details of the steps in the construction of the PPIN were performed as described previously^54^. Briefly, lipocalins were used as seed proteins and mapped to the parental PPIN. Cytoscape menus of “Select → Nodes → First Neighbors of Selected Nodes” and “New → Network → From Selected Nodes, All Edges” were used to extract the PPIN. Only the first level of interactions was extracted, generating the specific lipocalin PPIN. Duplicated edges, single nodes, and self-interactions in the lipocalin PPIN were considered redundant and removed.

Gene ontology (GO) annotation was integrated into the lipocalin PPIN by mining for enriched GO “biological process” terms of proteins using the ClueGO plugin, which facilitates the annotation and visualization of enriched GO terms in the form of network. In this analysis, only the enriched GO terms with *P*-values < 0.01 were considered significant. A kappa score was set to 0.3 as the threshold, indicating the relationships between the terms based the number of same genes^55^.

### Expression of lipocalins in esophageal carcinoma

The esophageal carcinoma expression profile GSE26886 is available at GEO (http://www.ncbi.nlm.nih.gov/geo/), which detects the expression data from 19 normal esophageal squamous epithelial samples, 21 esophageal adenocarcinoma (EAC) samples, and 9 esophageal squamous cell carcinoma samples (ESCC)^56^. The fold changes of the expression of genes in EAC and ESCC compared to normal esophageal squamous epithelium and analyzed using the GEO2R program^57^. The fold-changes of lipocalins and their interacting proteins in EAC and ESCC served as parameters were displayed in PPIN.

### Network topological parameters analyses

The topological parameters of lipocalins PPIN were analyzed using NetworkAnalyzer, which can compute computing network diameter, density, centralization, heterogeneity, and clustering coefficient, providing insight into the organization and structure of complex networks^58^. The degree of a node is the number of proteins in the network to which it is directly connected. In this study, the power law of distribution of node degrees, one of most important network topological characteristics, was analyzed as in our previous works^54^. Briefly, distribution of node degree *P*(*k*) is defined as the number of nodes with a degree *k* for *k *= 0, 1, 2, … The pattern of their dependencies was determined by fitting a line on the node degree distribution data. NetworkAnalyzer calculates the fitting the line where the power law curve of the forms y = βx^a^. The R^2^ value is used to quantify the fit to the power line, which is very close to 1 when the fit is good.

### Subcellular layers of the lipocalin PPIN

The subcellular localization classification of each protein in the lipocalins PPIN was retrieved from the HPRD GENE-ONTOLOGY annotation file, which was imported into Cytoscape as a node attribute of lipocalin PPIN. Cerebral (http://www.pathogenomics.ca/cerebral/) was used to re-distribute the nodes in lipocalins PPIN into different subcellular localizations without changing their interactions, which looks like a pathway diagram^59^. The igraph R program was used to find the shortest path between LCN2 and RB1 (retinoblastoma 1) in the lipocalin PPIN. The shortest path algorithm was able to find the shortest connection between two nodes in the graph^60^. The protein members in these shortest paths were also displayed in different layouts according to their subcellular localizations, showing the possible pathways from LCN2 to RB1.

### Expression correlation of lipocalin and their interacting proteins in esophageal carcinoma

The expression correlations between every two lipocalins in esophageal carcinoma, including EAC and ESCC, were analyzed by a customized R program using Pearson correlation method. The correlation coefficient was log-transformed and clustered using Cluster 3.0 software and viewed in TreeView^49^. Moreover, to gain a full view of their correlation patterns, the expression correlation for every two interacting protein was integrated into the lipocalin PPIN and served as edge weight.

## Additional Information

**How to cite this article**: Du, Z.-P. *et al*. A systematic analysis of human lipocalin family and its expression in esophageal carcinoma. *Sci. Rep*. **5**, 12010; doi: 10.1038/srep12010 (2015).

## Supplementary Material

Supplementary Information

## Figures and Tables

**Table 1:** Human lipocalins.

Official Symbol	Official Full Name	GeneID	Chromosome Location
AMBP	alpha-1-microglobulin/bikunin precursor	259	9q32-q33
APOD	apolipoprotein D	347	3q29
APOM	apolipoprotein M	55937	6p21.33
C8G	complement component 8, gamma polypeptide	733	9q34.3
CRABP1	cellular retinoic acid binding protein 1	1381	15q24
CRABP2	cellular retinoic acid binding protein 2	1382	1q21.3
FABP1	fatty acid binding protein 1, liver	2168	2p11
FABP12	fatty acid binding protein 12	646486	8q21.13
FABP2	fatty acid binding protein 2, intestinal	2169	4q28-q31
FABP3	fatty acid binding protein 3, muscle and heart (mammary-derived growth inhibitor)	2170	1p33-p32
FABP4	fatty acid binding protein 4, adipocyte	2167	8q21
FABP5	fatty acid binding protein 5 (psoriasis-associated)	2171	8q21.13
FABP6	fatty acid binding protein 6, ileal	2172	5q33.3-q34
FABP7	fatty acid binding protein 7, brain	2173	6q22-q23
FABP9	fatty acid binding protein 9, testis	646480	8q21.13
LCN1	lipocalin 1	3933	9q34
LCN10	lipocalin 10	414332	9q34.3
LCN12	lipocalin 12	286256	9q34.3
LCN15	lipocalin 15	389812	9q34.3
LCN1P1	lipocalin 1 pseudogene 1	286310	9q34.2
LCN2	lipocalin 2	3934	9q34
LCN6	lipocalin 6	158062	9q34.3
LCN8	lipocalin 8	138307	9q34.3
LCN9	lipocalin 9	392399	9q34.3
LCNL1	lipocalin-like 1	401562	9q34.3
OBP2A	odorant binding protein 2A	29991	9q34
OBP2B	odorant binding protein 2B	29989	9q34
ORM1	orosomucoid 1	5004	9q32
ORM2	orosomucoid 2	5005	9q32
PAEP	progestagen-associated endometrial protein	5047	9q34
PMP2	peripheral myelin protein 2	5375	8q21.3-q22.1
PTGDS	prostaglandin D2 synthase 21kDa (brain)	5730	9q34.2-q34.3
RBP1	retinol binding protein 1, cellular	5947	3q23
RBP2	retinol binding protein 2, cellular	5948	3q23
RBP4	retinol binding protein 4, plasma	5950	10q23.33
RBP5	retinol binding protein 5, cellular	83758	12p13.31
RBP7	retinol binding protein 7, cellular	116362	1p36.22

**Table 2:** Significant correlations in the expression of lipocalins in esophageal carcinoma.

EAC		ESCC	
Lipocalin	Highly co-expressed lipocalins	lipocalin	Highly co-expressed lipocalins
C8G	CRABP1, LCN1, FABP4, PAEP OBP2B, LCN15, PMP2, OBP2A, ORM1, RBP5, LCN6, FABP7	OBP2B	LCN1, PAEP, OBP2A; LCN8, LCN1, LCN15, LCNL1, CRABP1, RBP5, ORM1
CRABP1	C8G, LCN1, FABP4, PAEP OBP2B, LCN15, PMP2, OBP2A, ORM1, RBP5, LCN6, FABP7, LCNL1, LCN8, FABP3	LCN1	PAEP, OBP2A; LCN8, OBP2B, LCN1, LCN15, LCNL1, CRABP1, RBP5, ORM1
LCN1	FABP4, PAEP OBP2B, LCN15, PMP2, OBP2A, ORM1, RBP5, LCN6, LCN12, FABP7, LCNL1, LCN8, FABP3, RBP2	PAEP	OBP2A; LCN8, OBP2B, LCN1, LCN15, LCNL1, CRABP1, RBP5, ORM1
FABP4	PAEP, OBP2B, LCN15, PMP2, OBP2A, ORM1, RBP5, PMP2, OBP2A, ORM1, RBP5, LCN6, LCN12, FABP7, LCNL1, FABP3	OBP2A	LCN8, OBP2B, LCN1, LCN15, LCNL1, PAEP, CRABP1, RBP5, ORM1
PAEP	C8G, LCN1, FABP4, PAEP OBP2B, LCN15, PMP2, OBP2A, ORM1, RBP5, FABP7, LCNL1, FABP3	LCNL1	PAEP: OBP2A; LCN8, OBP2B, LCN1, LCN15, CRABP1, RBP5
		LCN8	OBP2B, LCN1, LCN15, LCNL1, PAEP, OBP2A, RBP5
		LCN2	RBP4, APOD

**Table 3:** Possible signal pathways from LCN2 to RB1.

No.	Proteins of the path								
1	LCN2	→	HGF	→	FN1	→	RBBP4	→	RB1
2	LCN2	→	HGF	→	FN1	→	SUMO2	→	RB1
3	LCN2	→	HGF	→	FN1	→	HDAC1	→	RB1
4	LCN2	→	HGF	→	FN1	→	UBC	→	RB1
5	LCN2	→	LRP2	→	RBP1	→	SIRT1	→	RB1
6	LCN2	→	LRP2	→	RBP1	→	RBBP7	→	RB1
7	LCN2	→	LRP2	→	RBP1	→	HDAC2	→	RB1
8	LCN2	→	LRP2	→	APP	→	MORF4L1	→	RB1
9	LCN2	→	LRP2	→	APP	→	PPARG	→	RB1
10	LCN2	→	LRP2	→	APP	→	CCND3	→	RB1
11	LCN2	→	LRP2	→	RBP1	→	RBBP4	→	RB1
12	LCN2	→	LRP2	→	RBP1	→	HDAC1	→	RB1
13	LCN2	→	LRP2	→	APP	→	UBC	→	RB1
14	LCN2	→	LRP2	→	SERPINE1	→	UBC	→	RB1
15	LCN2	→	LRP2	→	ALB	→	UBC	→	RB1
16	LCN2	→	LRP2	→	RBP1	→	UBC	→	RB1
17	LCN2	→	MMP9	→	FN1	→	RBBP4	→	RB1
18	LCN2	→	MMP9	→	FN1	→	SUMO2	→	RB1
19	LCN2	→	MMP9	→	FN1	→	HDAC1	→	RB1
20	LCN2	→	MMP9	→	FN1	→	UBC	→	RB1
