# Supplementary material for: A systematic analysis of human lipocalin family and its expression in esophageal carcinoma
Source: Sci Rep. 2015 Jul 1;5:12010. doi: 10.1038/srep12010 (PMC4487233; doi:10.1038/srep12010)
Supplement: Supplementary Information [file srep12010-s1.doc]

**A systematic analysis of human lipocalin family and its expression in esophageal carcinoma**

Ze-Peng Du^1^*, Bing-Li Wu^2^*, Xuan Wu^1^, Xuan-Hao Lin^1^, Xiao-Yang Qiu^1^, Xiao-Fen Zhan^1^, Shao-Hong Wang^1^, Jin-Hui Shen^1^, Chun-Peng Zheng^3^, Zhi-Yong Wu^3^, Li-Yan Xu^4^,*, Dong Wang^5^, En-Min Li^2^

^1^ Department of Pathology, Shantou Central Hospital, Affiliated Shantou Hospital of Sun Yat-sen University, Shantou515041, China; ^2^ Department of Biochemistry and Molecular Biology, Shantou University Medical College, Shantou 515041, China; ^3^ Department of Oncology Surgery, Shantou Central Hospital, Affiliated Shantou Hospital of Sun Yat-sen University, Shantou515041, China; ^4^ Institute of Oncologic Pathology, Shantou University Medical College, Shantou 515041, China; ^5^ College of Bioinformatics Science and Technology, Harbin Medical University, Harbin150000, China

*These authors contributed equally to this work

Correspondence and requests for materials should be addressed to B.W. ([blwu@stu.edu.cn](mailto:blwu@stu.edu.cn)) or l.X. ([lyxu@stu.edu.cn](mailto:nmli@stu.edu.cn)) (Fax: +86-754-88900847;Tel: +86-754- 88900413)

**Supplementary Figure S1**

**
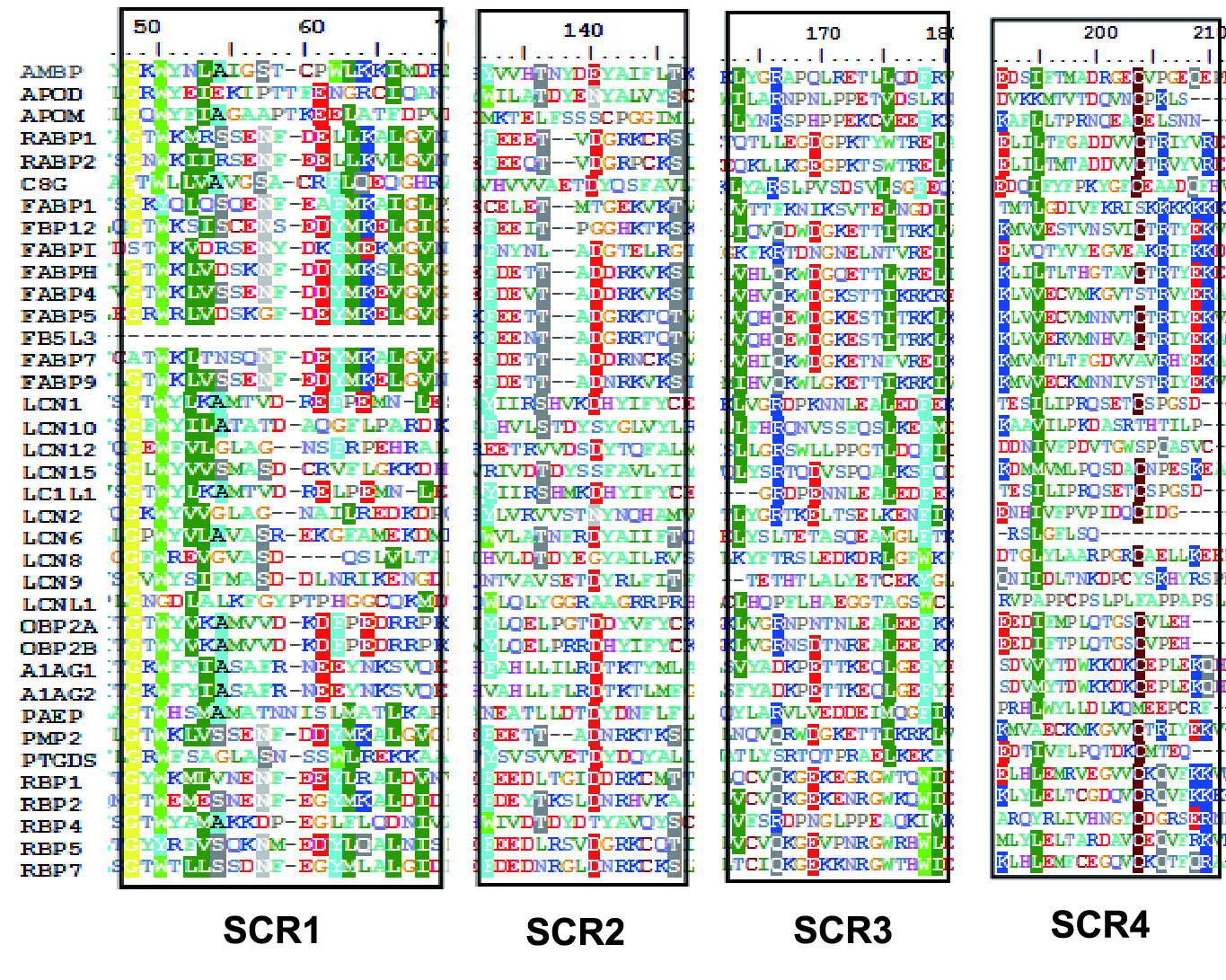
**

**Supplementary Figure S1**: Four structurally conserved regions are found in lipocalin family protein sequences.

**Supplementary Figure S2**

**
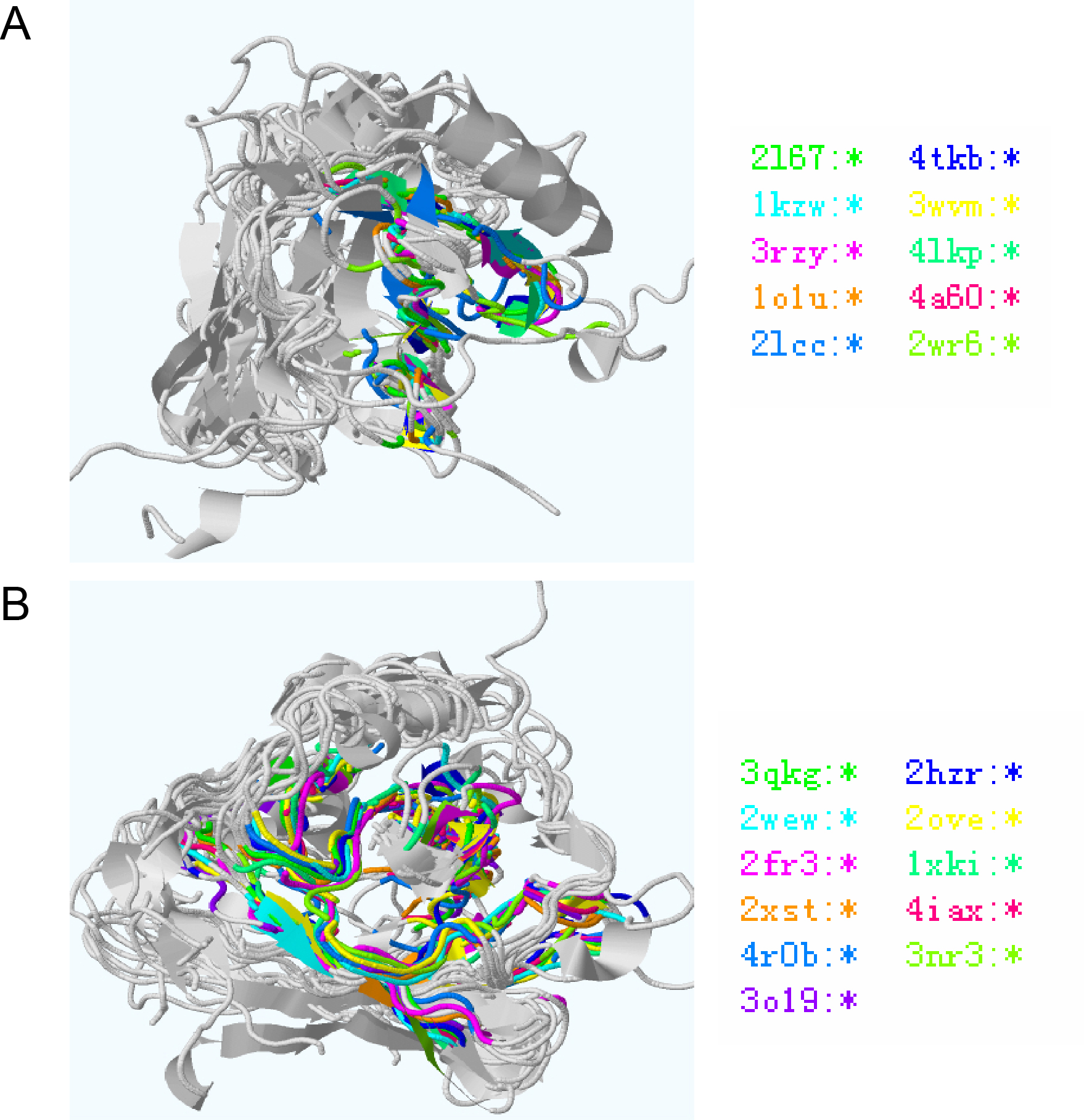
**

**Supplementary Figure S2**: Comparison the 3-dimensional structures of two grouped lipocalins. (A) The first grouped of lipocalin contains RABPs, RBPs, and FABPs. (B) The second grouped of lipocalins includes A1AGs, LCNs, OBP2s and others. The currently available structures of lipocalins were obtained from the PDB database and compared using PDBeFold.

**Supplementary Figure S3**


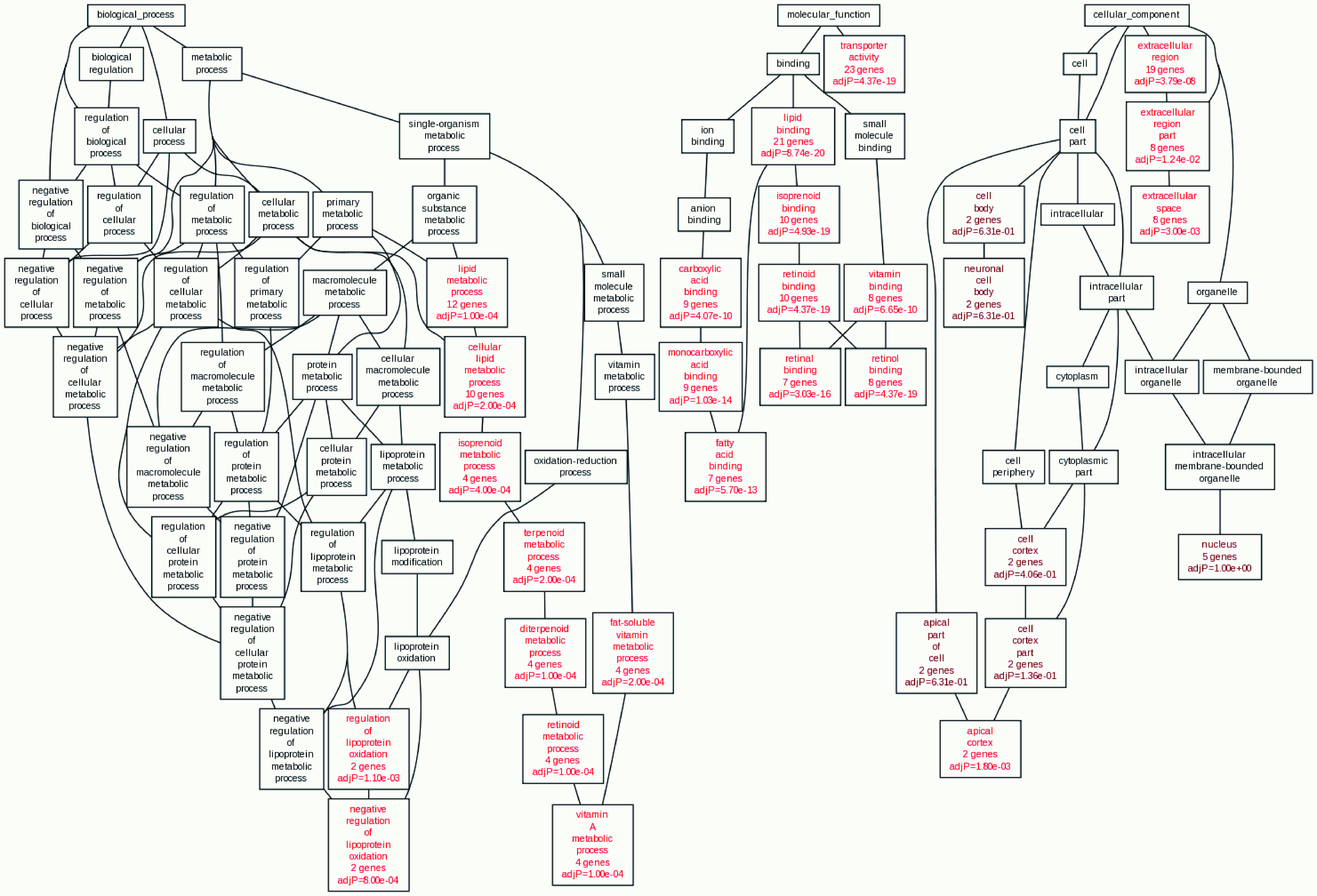


**Supplementary Figure S3:** Gene Ontology (GO) enrichment analysis of human lipocalins was performed using WebGestalt, which contains information from Gene Ontology Tree Machine software (<http://bioinfo.vanderbilt.edu/webgestalt/>). The whole human genome gene set was set as a reference list. The hypergeometric statistical method test was used, and only statistically enriched terms (*P* < 0.05) with at least 2 genes were selected. Significant GO enrichment results are indicated in red.

**Supplementary Figure S4**

**
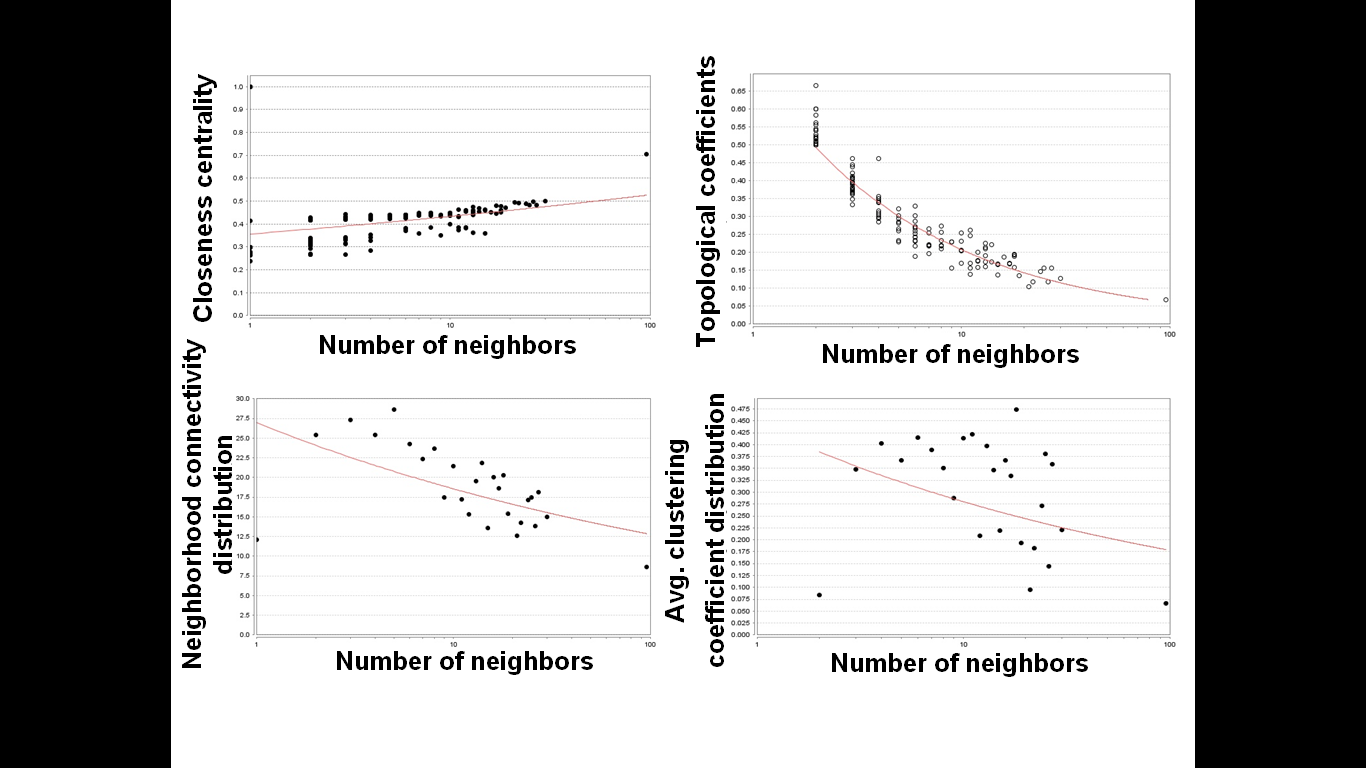
**

**Supplementary Figure S4**: Four important network topology parameters of lipocalin PPIN, including closeness centrality, topological coefficients, neighborhood connectivity distribution and average clustering coefficient distribution, were analyzed by NetworkAnalyzer plugin.

**Supplementary Figure S5:**


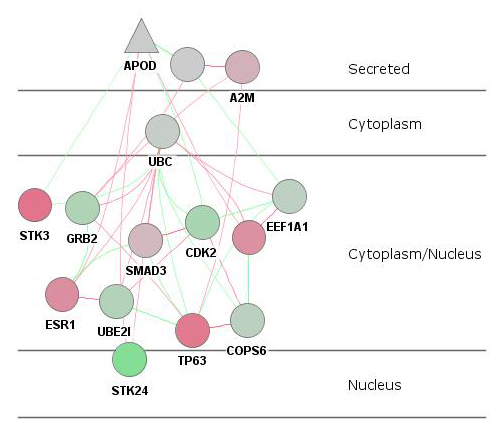


**Supplementary Figure S5:** Seventeen possible paths from APOD to TP63 integrated with their subcellular localization.

| **Table S1.** Currently available three-dimensional structures of lipocalins | | | |
| --- | --- | --- | --- |
| **Official Symbol** | **PDB ID** | **Resolution (**Å**)** | **Residue Count** |
| AMBP | 3QKG | 2.3 | 193 |
| APOD | 2HZR | 1.80 | 174 |
| APOM | 2WEW | 1.95 | 172 |
| C8G | 2OVE | 2.00 | 182 |
| CRABP2 | 2FR3 | 1.48 | 137 |
| FABP1 | 2L67 | 1.8 | 126 |
| FABP12 | 4TKB | 0.86 | 133 |
| FABP2 | 1KZW | Solution NMR | 131 |
| FABP3 | 3WVM | 0.88 | 133 |
| FABP4 | 3RZY | 1.08 | 139 |
| FABP5 | 4LKP | 1.67 | 138 |
| FABP6 | 1O1U | 1.8 | 127 |
| FABP9 | 4A60 | 1.53 | 154 |
| LCN1 | 1XKI | 1.80 | 162 |
| LCN15 | 2XST | 1.63 | 161 |
| LCN2 | 4IAX | 1.90 | 188 |
| ORM1 | 3KQ0 | 1.80 | 192 |
| PAEP | 4R0B | 2.45 | 169 |
| PMP2 | 3NR3 | 1.95 | 153 |
| PTGDS | 3O19 | 1.66 | 162 |
| RBP1 | 2LCC | Solution NMR | 76 |
| RBP4 | 2WR6 | 1.80 | 175 |

| **Table S2.** Possible signal pathways from APOD to TP63. | | | | | | | |
| --- | --- | --- | --- | --- | --- | --- | --- |
| No. | Proteins of the path | | | | | | |
| 1 | APOD | → | SHBG | → | A2M | → | TP63 |
| 2 | APOD | → | ESR1 | → | GRB2 | → | TP63 |
| 3 | APOD | → | SHBG | → | GRB2 | → | TP63 |
| 4 | APOD | → | CDK2 | → | COPS6 | → | TP63 |
| 5 | APOD | → | CUL2 | → | COPS6 | → | TP63 |
| 6 | APOD | → | CDK2 | → | EEF1A1 | → | TP63 |
| 7 | APOD | → | SHBG | → | EEF1A1 | → | TP63 |
| 8 | APOD | → | CUL2 | → | EEF1A1 | → | TP63 |
| 9 | APOD | → | CDK2 | → | SMAD3 | → | TP63 |
| 10 | APOD | → | ESR1 | → | SMAD3 | → | TP63 |
| 11 | APOD | → | CDK2 | → | UBC | → | TP63 |
| 12 | APOD | → | ESR1 | → | UBC | → | TP63 |
| 13 | APOD | → | STK24 | → | UBC | → | TP63 |
| 14 | APOD | → | STK3 | → | UBC | → | TP63 |
| 15 | APOD | → | CUL2 | → | UBC | → | TP63 |
| 16 | APOD | → | CDK2 | → | UBE2I | → | TP63 |
| 17 | APOD | → | ESR1 | → | UBE2I | → | TP63 |
